# The Evaluation of Different Treatment Approaches in Patients With Earthquake-Related Crush Syndrome

**DOI:** 10.7759/cureus.47194

**Published:** 2023-10-17

**Authors:** Sümeyra Koyuncu, Hilal Sipahioglu, Oğuzhan Bol, Hatice Kübra Zenger İlik, Aslıhan Dilci, Merve Elmaağaç, Merve Yalçınkaya, Vedat Gencer, Fırat Ozan, Ali İhsan Günal, Ismail Kocyigit

**Affiliations:** 1 Nephrology, Kayseri State Hospital, Kayseri, TUR; 2 Internal Medicine, Kayseri City Training and Research Hospital, Kayseri, TUR; 3 Emergency Medicine, University of Health Sciences, Kayseri City Hospital, Kayseri, TUR; 4 Internal Medicine, Kayseri City Hospital, Kayseri, TUR; 5 Orthopaedics, Kayseri City Hospital, Kayseri, TUR; 6 Nephrology, Erciyes Universty, Kayseri, TUR; 7 Nephrology, Erciyes University, Kayseri, TUR

**Keywords:** maras, acute renal fail, hemodialysis, turkey-syria earthquake, crush syndrome

## Abstract

Background: On February 6, 2023, an earthquake occurred in Kahramanmaras, Turkey, resulting in loss of life, injuries, and the displacement of thousands of people. The aim of this study is to determine the factors affecting amputation and fasciotomy decisions in patients with crush syndrome, along with clinical laboratory parameters.

Materials and methods: The study included patients over 18 years of age who presented with crush injuries and exhibited systemic symptoms. Inclusion criteria comprised patients with creatine kinase (CK) levels exceeding 1,000 IU/L, oliguria (urine output less than 400 mL per day), elevated blood urea nitrogen (BUN) levels surpassing 40 mg/dL, serum creatinine exceeding 1.5 mg/dL, potassium levels over 6 mEq/L, phosphorus levels surpassing 8 mg/dL, and serum calcium levels below 8 mg/dL. Multiple parameters were evaluated, including blood glucose, serum sodium, potassium, calcium, phosphorus, BUN, creatinine, uric acid, CK, albumin, alanine aminotransferase (ALT), aspartate aminotransferase (AST), total bilirubin, direct bilirubin, prothrombin time (international normalized ratio (INR)), urinalysis, C-reactive protein (CRP), venous blood gas, ECG, and chest radiography.

Results: Following the Maraş earthquake, a total of 3,184 patients were admitted to our hospital within the first seven days. Out of these patients, 2,216 received outpatient treatment, 639 were hospitalized in the general ward, and 128 were admitted to the intensive care unit. Among the admitted patients, 237 were diagnosed with crush syndrome, with 126 being male and 111 being female. The average duration of being trapped under debris was eight hours, ranging from four to 36 hours.

In the study population, extremity trauma was observed in 84 patients, thoracic trauma in 32 patients, and abdominal trauma in 20 patients. Erythrocyte replacement was administered to 123 patients, while fresh frozen plasma was given to 69 patients, for a total of 1008 units utilized. Mannitol infusions were provided to 58 patients, while bicarbonate infusions were administered to 116 patients. Among the cohort, 71 patients underwent dialysis, with nine of them receiving hemodialysis along with mannitol. Additionally, 67 patients experienced stage 3 acute kidney injury, and 41 patients were deceased. None of the patients required permanent hemodialysis.

Conclusion: Earthquakes are considered to be one of nature’s most significant and inevitable disasters. While it is impossible to prevent them, effective management strategies are crucial in mitigating the ensuing chaos and reducing casualties. In order to achieve this, it is imperative to draw lessons from past seismic events and apply appropriate treatment protocols to the affected individuals.

## Introduction

On February 6, 2023, two massive earthquakes, measuring 7.7 and 7.6 on the Richter scale, struck the Kahramanmaras province of Turkey nine hours apart, resulting in injuries, deaths, and the displacement of thousands. The first earthquake struck suddenly, catching people in their sleep and leaving them with no chance to escape. Tragically, this disaster resulted in over 40,000 fatalities. In the aftermath of these earthquakes, the injured were rescued in the hours and days that followed. The impact of the earthquake led to severe traumatic injuries and crush syndrome, and these injured individuals received treatment at the nearest healthcare facilities [1,2].

Complications with potentially life-threatening consequences, such as crush syndrome and acute kidney injury (AKI), pose significant medical challenges for victims of disasters. Among these complications, AKI stands out as a particularly critical concern that clinicians must address [3,4]. Early intervention for AKI is pivotal in reducing both mortality and morbidity associated with crush syndrome. Various studies have reported that up to 75% of AKI patients require hemodialysis. As a result, delivering timely and effective fluid therapy is of paramount importance in the treatment of crush syndrome, with immediate administration following the rescue and extraction of injured individuals from under debris being a critical step [5,6].

The outcomes of devastating earthquakes are influenced by various factors, including the type of trauma, comorbid events, and complications observed during the clinical course. Additionally, epidemiologic characteristics such as age, distance to reference hospitals, and the time between the disaster and admission to reference hospitals can also affect the outcomes. Serious electrolyte disorders, such as hyperkalemia, the need for dialysis, and other factors, can impact mortality rates [7,8]. In this study, our objective is to identify the factors that influence decisions regarding amputation and fasciotomy in patients with crush syndrome and examine potential correlations with clinical laboratory parameters.

## Materials and methods

This study received approval from the Ethics Committee of Kayseri City Hospital (approval no. 2023/839). Throughout the study, the investigators upheld patient confidentiality and adhered to the ethical principles of clinical trials as outlined in the Declaration of Helsinki.

Following the earthquake, approximately 4000 patients were admitted to our hospital within the first seven days. After initial evaluation in the emergency department, patients diagnosed with crush syndrome underwent further assessment by nephrology specialists. Systemic manifestations that are induced by crush injury are referred to as crush syndrome. Most patients received fluid therapy during transportation from the earthquake zone, which was promptly continued upon arrival at our hospital, located approximately 270 km away from the nearest earthquake zone. To ensure appropriate fluid replacement, the initial fluid given during transfer and the baseline urine output at admission were taken into consideration. Subsequently, fluid replacements were adjusted based on the urine output after reaching 3000 ml. If there was urine output, a continuous infusion of 100 ml of 0.9% sodium chloride (NaCl) per hour was maintained. In all suitable patients, a urinary catheter was inserted for monitoring purposes, and close follow-up was conducted.

The parameters assessed in this study included blood glucose levels, serum sodium, potassium, calcium, phosphorus, blood urea nitrogen (BUN), creatinine, uric acid, creatinine kinase (CK), albumin, alanine aminotransferase (ALT), aspartate aminotransferase (AST), total bilirubin, direct bilirubin, prothrombin time (international normalized ratio (INR)), urinalysis, C-reactive protein (CRP), venous blood gas analysis, ECG, and chest radiography. The data obtained from these evaluations was meticulously recorded for further analysis.

Patients requiring immediate surgical intervention, such as a fasciotomy or amputation, were promptly taken to the operating room. Additionally, all patients received tetanus prophylaxis to prevent potential complications. Depending on their clinical condition, patients were monitored and cared for either in intensive care units or general wards. For patients requiring dialysis, central catheters were inserted to facilitate the procedure.

The study included patients aged 18 years or older who presented with crush injuries accompanied by systemic symptoms. Patients with a CK level greater than 1000 IU/L, oliguria (urine output less than 400 mL/day), elevated BUN levels (>40 mg/dL), serum creatinine levels above 1.5 mg/dL, potassium levels exceeding 6 mEq/L, phosphorus levels above 8 mg/dL, or serum calcium levels below 8 mg/dL were included in the study. Patients under the age of 18, individuals who did not meet the specified laboratory inclusion criteria, as well as patients who received outpatient treatment in the emergency department and were discharged on the same day, were excluded from the study.

Statistical analysis

All statistical analyses were performed using SPSS Statistics version 22.0 (IBM Corp., Armonk, NY, USA). Continuous variables with a normal distribution were presented as the mean ± SD, and continuous variables with a skewed distribution were expressed as the median (range). Categorical variables were reported as numbers (n) and percentages (%). A forward stepwise binary logistic regression was conducted with variables having a p-value of less than 0.1 in univariate analysis to determine independent factors predicting fasciotomy and amputation. Results are presented as odds ratios (OR) and confidence intervals (CI).

## Results

As a result of the earthquake, our city did not experience significant damage. However, due to the proximity of the healthcare facility, it received a high number of referrals, with thousands of patients seeking medical attention. During the first seven days following the earthquake, a total of 3184 patients were admitted to our hospital. Among them, 3032 patients were over 18 years old. Of these, 2216 were treated as outpatients, 639 were admitted to the general ward, and 128 were placed in the intensive care unit. Among the patients, 237 were diagnosed with crush syndrome, with 126 males and 111 females (Table 1). On average, patients were trapped under debris for approximately eight hours, ranging from four to 36 hours.

**Table 1:** Clinical and demographic information of crush syndrome patients who underwent fasciotomy and amputation AKI: Acute kidney injury, CRP: C-reactive protein, CK: Creatinine kinase, BUN: Blood urea nitrogen, Hgb: Hemoglobin, LDH: Lactate dehydrogenase, AST: Aspartate aminotransferase, ALT: Alanine aminotransferase, HCO3: Bicarbonate

Variables	Fasciotomy (n = 70)	Hyperbaric oxygen therapy (n = 33)	Amputation (n = 32)
Average time under the rubble (hour), n (%)	14 (7-59)	58 (29-72)	52 (12-72)
AKI Stage 1, n (%)	5 (7.1)	3 (9)	13 (40.6)
AKI Stage 2, n (%)	4 (5.7)	2 (6)	7 (21.8)
AKI Stage 3, n (%)	36 (51.4)	10 (30.3)	9 (23.2)
Hemodialysis, n (%)	39 (55.7)	9 (27.2)	18 (54.5)
CRP (mg/dL)	113 (74-155)	122 (78.5-156)	94 (75-132)
CK (U/L)	40,496(10,517-86,623)	15,495 (7550-57,636)	17,192 (8076-77,147)
BUN (mg/dl)	38 (18-54)	24 (9-49)	35 (17-54)
Creatinine (mg/dl)	2.3 (0.6-3.5)	0.6 (0.5-2.4)	1.4 (0.6-3.4)
Sodium (mmol/l)	135 (130-139)	138 (135-142)	137 (132-142)
Chloride (mmol/l)	101 (98-107)	105 (101-110)	104 (101-111)
Potassium (mmol/l)	5.1 (4.1-5.9)	4.3 (3.7-5.3)	4.9 (4.1-6.0)
Calcium (mg/dl)	6.8(6-7.5)	7.2(6.6-7.6)	6.7(5.9-7.8)
Phosphorus (mg/dl)	5 (3.1-6.4)	3.2 (2.3-5)	4.3 (3-6)
Albumin (g/L)	2.6±0.6	2.5 (2.4-3)	2.4 (2.1-2.9)
Hgb (g/dL)	12±3.4	12.3±2.3	12±2.8
Leukocyte (x109/L)	14 (11-20)	14.3 (11.6-19)	12 (9-17)
Neutrophils (x109/L)	12,150 (9237-17,100)	12,100 (9250-16,700)	13,100 (9675-19,485)
Lymphocytes (x109/L)	1475 (1052-1977)	1110 (865-1720)	1495 (1010-2625)
Platelets (x109/L)	200±78	197 (171-243)	216±76
Uric acid (mg/dl)	5.2 (3.2-7)	4.6 (3.1-6.7)	57 (3.7-7)
LDH (U/L)	1451 (734-2570)	835 (576-1 =451)	1003 (555-2863)
AST (U/L)	613 (269-1025)	265 (163-643)	421 (169-980)
ALT (U/L)	229 (119-367)	123 (85-230)	177 (95-400)
HCO3 (mmol/L)	19.6±4.6	21 (17-24)	18 (16-23)

Regarding specific injuries, 84 patients had extremity trauma, 32 had thoracic trauma, and 20 had abdominal trauma. A total of 123 patients received erythrocyte replacement, while 69 patients received fresh frozen plasma replacement, amounting to a total of 1008 units transfused.

Out of the total number of patients, 84 had extremity trauma, 32 had thoracic trauma, and 20 had abdominal trauma. Among the treatment measures taken, mannitol infusion was administered to 58 patients, while bicarbonate infusion was given to 116 patients. Additionally, 71 patients required dialysis, with nine of them specifically undergoing hemodialysis with mannitol. Among the patients, 67 were diagnosed with stage 3 AKI. Tragically, 41 patients did not survive and passed away.

Most patients presented with elevated CRP levels and showed a tendency toward hyponatremia. Additionally, low levels of albumin were observed, while lactate dehydrogenase (LDH), AST, and ALT values were elevated. Table 2 provides details of the laboratory parameters of the patients during their initial visit to the emergency department.

**Table 2:** Demographic and transfusion data of patients with crush syndrome HCO3: Bicarbonate, AKI: Acute kidney injury, HD: Hemodialysis, FFP: Fresh frozen plasma

Variables	Patient n (%), Total n = 237
Age (year, ±sd)	34±12.4
Gender: F	111 (46.8)
The average time under the rubble (hour) median (IQR)	8 (4-36)
Comorbidities	
Hypertension	23 (9.7)
Diabetes Mellitus	11 (4.6)
Coronary artery disease	2 (0.8)
Chronic kidney disease	3 (1.2)
Other	56 (23.6)
Extremity Trauma	84 (35.8)
Abdominal Trauma	20 (8.4)
Thoracic Trauma	32 (13.6)
Erythrocyte transfusion	123 (51.8)
Albumin infusion	1 (0.4)
Mannitol infusion	58 (24.4)
HD	71 (29.9)
HCO3 infusion	116 (48.9)
Mannitol via HD	9 (3.7)
FFP	69 (29.1)
Erythrocytes	123 (51.8)
AKI	
Stage 1	13 (5.4)
Stage2	7 (2.9)
Stage 3	67 (28.2)
Mortality	41 (17.2)

Within the cohort of patients diagnosed with crush syndrome, a total of 70 individuals underwent fasciotomy, 32 required amputation surgery, and 33 received hyperbaric oxygen therapy. The need for amputation was found to be directly proportional to the duration of time spent under debris. Additionally, the requirement for hemodialysis was more prevalent among patients who underwent fasciotomies. Table 3 offers a comparison of patients who underwent fasciotomy, amputation, and hyperbaric oxygen therapy.

**Table 3:** Laboratory findings of crush syndrome CRP: C-reactive protein, CK: Creatinine kinase, BUN: Blood urea nitrogen, Cl: Chloride, Hgb: Hemoglobin, LDH: Lactate dehydrogenase, AST: Aspartate aminotransferase, ALT: Alanine aminotransferase, HCO3: Bicarbonate

Variables	% / Mean ± SD, median (IQR)
CRP (mg/dL)	88 (44-146)
CK (U/L)	8628 (2702-41,998)
BUN (mg/dl)	20 (11-42)
Creatinine (mg/dl)	0.8 (0.6-2.4)
Sodium (mmol/l)	136.9 ±6
Cl (mmol/l)	102±10.8
Potassium (mmol/l)	4.5±0.9
Calcium (mg/dl)	7.5±1.1
Phosphorus (mg/dl)	3.3 (2.6-4.8)
Albumin (g/L)	3.2 (2.6-3.7)
Hgb (g/dL)	12±2.8
Leukocyte (x109/L)	12,000 (9000-17,000)
Lymphocytes (x109/L)	1500(1055-2040)
Platelets (x109/L)	216±76
Uric acid (mg/dl)	4.4 (3.2-6.3)
LDH (U/L)	628 (398-1 414)
AST (U/L)	198 (70-602)
ALT (U/L)	105 (39-237)
HCO3 (mmol/L)	21.2±4.3
Lactat (mmol/L)	1.4 (1.0-2.1)
Lymphocytes (x109/L)	1 500 (1055-2040)

The study aimed to investigate the association between laboratory parameters and transfusion requirements among patients, as well as explore any potential correlation between fasciotomy and amputation. To achieve this, multivariate analyses were conducted using the parameters identified as significant in the univariate analysis. These analyses revealed that patients who underwent fasciotomy exhibited a higher frequency of hyponatremia, hypoalbuminemia, and elevated phosphorus levels. The impact of various variables on the occurrence of fasciotomy and amputation in patients with crush injuries was analyzed using both univariate and multivariate analyses.

A total of 71 patients with AKI underwent hemodialysis. Among them, 53 hemodialysis sessions were performed on the first day, 96 sessions on the second day, and a total of 810 sessions were performed within one month due to the earthquake. The most common indications for hemodialysis were hyperkalemia and metabolic acidosis. None of the patients required long-term hemodialysis treatment.

## Discussion

Sixty years ago, Bywaters defined crush syndrome as the clinical picture of acute renal failure developing in war victims who experienced crush trauma and subsequently died [9]. Crush syndrome, also known as traumatic rhabdomyolysis and AKI encompasses symptoms characterized by ischemic necrosis of muscle tissue due to prolonged gravitational compression of the limbs or trunk, along with electrolyte imbalances [10-12].

The incidence of crush syndrome varies among different regions. In Tangshan, China, it was reported to be 2% to 5%; in Hanshin-Awai, Japan, 7.49%; and in Mexico City, 25.15%, compared to 18% in Marmara. These differences may be attributed to factors such as climate, construction material, duration of compression, availability, and efficiency of rescue teams and medical support, distance from the epicenter to reference hospitals, and challenges in data collection due to the conditions following a disaster [13-17].

In our study, we observed a high incidence of crush syndrome in hospitalized patients, with a rate of 37%. This elevated rate could potentially be attributed to the circumstances surrounding the earthquake, as many individuals were trapped during the night. Despite our hospital being located far away from the earthquake zone, a significant number of patients arrived with fluid therapy already initiated. Additionally, there were cases where patients managed to extract themselves from the debris and made their way to the hospital independently.

Acute kidney injury is one of the most fatal complications of CS [18]. After the Wenchuan earthquake in China, a study revealed that 41.6% of patients diagnosed with crush syndrome also developed AKI [19]. Similarly, in a separate investigation conducted by Sever et al., it was found that approximately 12% of the 5302 individuals injured in the Marmara earthquake in Turkey developed AKI due to crush syndrome [20]. Despite attempts to alleviate acute issues through dialysis treatment, the majority of patients ultimately succumb to multiple organ failure as a consequence of the systemic inflammatory response [21]. Within the context of our study, the mortality rate was determined to be 17.2%, with AKI being observed in 40% of patients. Out of the total cohort, 90 patients underwent at least one session of hemodialysis, but none of them necessitated definitive hemodialysis.

Due to advancements in dialysis technology, it has been observed that lives can be saved in appropriate cases and renal function can be fully restored within a short period [22-24]. After the Marmara earthquake, various dialysis methods, such as intermittent hemodialysis (IHD), continuous renal replacement therapy (CRRT), and peritoneal dialysis (PD), were employed to treat the affected patients. At our clinic, 90 individuals received IHD treatment, while five patients underwent CRRT for their renal care [25].

Following crush syndrome, the prompt implementation of fasciotomies and occasionally amputations is imperative to restore blood flow to the affected tissues, thereby preventing irreversible damage and ensuing disability. The early application of fasciotomy plays a crucial role in managing the injured extremities. However, it is necessary to acknowledge that this procedure may increase morbidity in later stages due to the potential risks of infection, sepsis, and heightened coagulopathy. Despite the increased need for blood and plasma transfusions in patients undergoing fasciotomy, long-term follow-ups indicate a relatively low risk of infection, with no exacerbation of kidney failure.

The underlying mechanism of crush syndrome involves a rapid elevation in myoglobin and potassium levels, leading to nephrotoxic effects and cardiac arrhythmias. In patients with AKI caused by crush syndrome, the implementation of a fasciotomy may pose a risk of morbidity and mortality due to potential complications such as bleeding, sepsis, and coagulopathy. However, a report from Bam, Iran, demonstrated that fasciotomy was carried out on 70 (35%) out of 200 patients with crush syndrome and AKI, with no observed increase in morbidity or mortality [26,27]. In the study, 70 patients underwent fasciotomy, 32 patients underwent amputation, and the fasciotomy group had a higher requirement for hemodialysis. Additionally, CRP levels were higher in the fasciotomy group, and more fresh frozen plasma (FFP) was needed for transfusion. Hyperbaric oxygen therapy was applied to 33 patients.

After the Marmara earthquake, there was a high demand for plasma, primarily for treating hemorrhagic diathesis caused by disseminated intravascular coagulation. Additionally, large volumes of human albumin were required, similar to blood and FFP transfusions. Multiple factors, such as leakage of plasma fluid from wounds in crush syndrome victims, malnutrition, fluid overload, and increased catabolic rate due to trauma, could contribute to hypoalbuminemia, which is known as a strong predictor of mortality in AKI [20,28]. Notably, patients who underwent fasciotomy had a higher incidence of hypoalbuminemia and hyponatremia compared to other treatment groups.

The Wenchuan earthquake revealed a common occurrence of hyponatremia in patients with crush syndrome, which is known to have a negative impact on the prognosis [29]. Our patients also exhibited a tendency toward developing hyponatremia and hypoalbuminemia, potentially attributable to fluid overload. During the Marmara earthquake, it was found that an average volume of approximately 5000 ml/day of fluids was administered. Although a high amount of fluid was given, some patients experienced hypervolemia, leading to the necessity of hemodialysis. Mannitol, which is purported to have various advantages for both renal and extrarenal functions [30,31], was administered to 58 patients. However, no clinically significant difference was observed in these patients. On the other hand, nine oliguric patients who were undergoing hemodialysis received mannitol before the treatment in order to enhance ultrafiltration. These patients were closely monitored in the intensive care unit. Previous studies have indicated that the duration of being trapped under debris and elevated levels of creatine phosphokinase (CPK), LDH, AST, and uric acid are the most significant predictive factors for the development of AKI [32]. It is worth noting that these values were higher in patients who required amputation.

Excessive swelling of the extremities, accompanied by a worsening of rhabdomyolysis, can manifest as a second-wave phenomenon of rhabdomyolysis, occurring between three and 12 days after the initial trauma [23,31]. Regular monitoring of abdominal and limb circumference on a daily basis can serve as an early predictor of impending complications. The exact reasons for fluid retention in this context are not fully understood. However, it is believed that the edema may be attributed to a combination of increased hydrostatic filtration pressure from vasodilated arterioles and capillaries, along with low intravascular capillary oncotic pressure due to hypoalbuminemia. Hypoalbuminemia is a common condition in patients with crush syndrome [33].

Our study had some limitations. The chaotic environment following the earthquake posed challenges in data collection and standardization. All patients could not be rescued from under the rubble in the same timeframe, and there was no standard treatment procedure.

## Conclusions

Earthquakes are considered to be one of nature’s most significant and inevitable disasters. While it is impossible to prevent them, effective management strategies are crucial in mitigating the ensuing chaos and reducing casualties. In order to achieve this, it is imperative to draw lessons from past seismic events and apply appropriate treatment protocols to the affected individuals.
